# The Hop-Pickers' Hospital Dilemma

**Published:** 1913-09-06

**Authors:** G. M. Livett

**Affiliations:** Vicar. Wateringbury Vicarage, Maidstone.


*To the Editor of* THE HOSPITAL.

### The Hop-pickers' Hospital Dilemma.

*To the Editor of* THE HOSPITAL.

SIR,—At Wateringbury, Kent, we have had, during the last twelve years, in connection with the Church of England Hop-pickers' Missionary Association, an improvised hospital for the stranger-pickers, numbering some thousands, who work in this and an adjoining parish. It contains three or four beds for children, and has an out-patients department in which from thirty to forty cases, more or less, are treated daily. It has been conducted by two resident nurses with a lady-help to do the cooking, etc., and the local doctor gives his services.

This year we have been unable to find either nurses or lady-help, and we have before us the heart-breaking prospect of being unable to open. Our last hope is that this appeal may meet the eye of ladies who will volunteer to come to the rescue. We have usually had two fully trained nurses, who, having given up their work as a profession, have been glad to revive it temporarily; but perhaps nurses in full work would be glad to spend their holiday in this way. A staff of one fully trained nurse and one assistant or Red Cross with a lady-help could do the work. Of course we pay all expenses. The hospital ought to be opened on the 8th inst., and the work continues for at least a fortnight. Communications may be addressed to me, and a personal interview, whereby, if necessary to save time, fuller particulars could be obtained, would be gladly accorded by the London representative of our Association, Miss Harvey, 39 Kempsford Gardens, S.W.

P.S.—A telegram just received from Miss Harvey says nurses are also wanted elsewhere in the hop-picking districts.

G. M. LIVETT, Vicar.

Wateringbury Vicarage, Maidstone.
September 1, 1913.

